# Pembrolizumab and lenvatinib in the treatment of recurrent ovarian carcinoma: A single institution experience

**DOI:** 10.1016/j.gore.2025.101811

**Published:** 2025-07-19

**Authors:** Helen Toma, Rebeca Kelly, Chelsea Katz, Hannah Hong, Hannah Diasti, David P. Warshal, Lauren Krill

**Affiliations:** a MD Anderson Cancer Center at Cooper University, Camden, NJ, USA; b Sidney Kimmel Cancer Center at Thomas Jefferson University Hospital, Philadelphia, PA, USA; c Cooper Medical School of Rowan University, Camden, NJ, USA

## Abstract

**Background:**

Advanced stage ovarian carcinoma has a poor prognosis with recurrence rates of over 80%, 5-year survival of 36–45%, and limited response to standard therapy. Pembrolizumab and lenvatinib are FDA approved for treatment of microsatellite stable (MSS)/mismatch repair proficient (pMMR) endometrial and renal cell cancers. Early phase II studies have shown promising results in a variety of advanced solid tumors, including ovarian cancer. We report on the clinical outcome of recurrent MSS/pMMR ovarian cancer patients treated with this therapy.

**Methods:**

For this retrospective cohort study, patients with a diagnosis of ovarian cancer treated with pembrolizumab and lenvatinib from January 2020 to April 2024 at MD Anderson Cancer Center at Cooper were identified. Demographic data, tumor characteristics, germline/somatic genetic testing, treatment duration, and toxicity were collected. Response rate by RECIST criteria, progression free survival (PFS), and clinical benefit rate were calculated.

**Results:**

Sixteen patients were identified. Most had high-grade serous (n = 11, 68.75 %) or clear cell histologies (n = 4, 25 %) and FIGO stage III/IV disease (n = 15, 93.75 %). Eighty-one percent had platinum resistant recurrent disease. Three patients discontinued therapy after one cycle, unrelated to drug toxicity, and were non-evaluable for response. Of 13 patients evaluable for response, 54 % had a partial response and 31 % had stable disease. The 6-month clinical benefit rate was 69 %. The median PFS for all evaluable patients was 7.9 months. At the time of data analysis, 2 patients remained on treatment.

**Conclusion:**

Pembrolizumab-lenvatinib therapy demonstrated favorable clinical benefit in recurrent, platinum resistant MSS/pMMR ovarian cancer, a group of patients in need of more therapeutic options.

## Highlights

• The use of single agent immune checkpoint inhibitors has not shown significant clinical benefit in ovarian cancer.
• Preclinical studies have shown that lenvatinib enhances antitumor activity of anti-PD-1 antibody.
• Pembrolizumab and lenvatinib are FDA approved for treatment of MSS/pMMR endometrial cancer and renal cell carcinoma.
• Pembrolizumab/lenvatinib is a safe, effective option for treatment of platinum resistant ovarian cancer.
• Reduced doses of lenvatinib may help mitigate toxicity while providing meaningful clinical response.

## 1 Introduction

Projections for 2025 estimate that 12,730 women will die of ovarian cancer in the United States (Siegel et al., 2025). Over half of diagnoses occur at advanced stages with 5 year survival rates of approximately 31 % (Siegel et al., 2024). While most patients respond well to first line therapy, typically a combination of cytoreductive surgery and platinum-based chemotherapy with or without PARP inhibitor use, over 70 % will recur and eventually develop treatment resistant disease (du Bois et al., 2009, St Laurent and Liu, 2024). Treatment of platinum resistant disease usually consists of single-agent cytotoxic therapies such as pegylated liposomal doxorubicin, gemcitabine, paclitaxel, and topotecan, with response rates between 10–15 % (St Laurent and Liu, 2024, Pujade-Lauraine et al., 2014, Kurzeder et al., 2016, Gaillard et al., 2021, Moore et al., 2021, Disis et al., 2019, Pujade-Lauraine et al., 2021) and a median progression free survival (PFS) of approximately 3.5 months (Mutch et al., 2007, Gordon et al., 2001, Sehouli et al., 2011). The phase 3 Aurelia trial demonstrated improved PFS with the addition of bevacizumab to standard single agent chemotherapy with no improvement in overall survival (OS) (Pujade-Lauraine et al., 2014). In light of these findings, there remains an unmet need for additional therapeutic options.

The rise of immunotherapy and targeted agents has led to remarkable outcomes for difficult to treat solid tumors such as non-small-cell lung cancer and metastatic melanoma. Despite ovarian cancer's high proportion of tumor infiltrating lymphocytes and its known use of the PD-L1 pathway to escape immune response (Abiko et al., 2013, Hamanishi et al., 2007), monotherapy immune checkpoint inhibitors (ICI) responses are low, ranging from 6-22 % (Brahmer et al., 2012, Varga et al., 2019, Matulonis et al., 2019, Infante et al., 2016). Other therapies including, more recently approved, mirvetuximab soravtansine and trastuzumab deruxtecan, can only be used in a subset of patients expressing appropriate markers (Meric-Bernstam et al., 2024, Immunogen, 2022).

Vascular endothelial growth factor (VEGF) is expressed in almost all ovarian tumors (van der Bilt, et al., 2012) and contributes to an immunosuppressive environment (Ziogas et al., 2012, Terme et al., 2013, Shrimali et al., 2010). Preclinical studies using murine models treated with a combination of an anti-PD-1 antibody and lenvatinib, a multiple receptor tyrosine kinase inhibitor, resulted in greater antitumor activity compared with anti-PD-1 treatment alone (Kato et al., 2019). Currently, pembrolizumab and lenvatinib are FDA approved for treatment of microsatellite stable (MSS)/mismatch repair proficient (pMMR) endometrial cancer and previously untreated advanced renal cell carcinoma. Early phase II studies and case series reports have shown promising treatment results in a variety of advanced solid tumors including ovarian malignancies (González-Martín et al., 2024, Calo et al., 2023, McNamara et al., 2023). Given these findings, we report on the clinical outcomes of recurrent ovarian cancer patients at our institution treated with this therapy.

## 2 Methods

This study was approved by the Cooper University Hospital Institutional Review Board (IRB#24–049). All patients with a diagnosis of ovarian cancer treated with pembrolizumab and lenvatinib from January 2020 to April 2024 at MD Anderson Cancer Center at Cooper, Camden, NJ were included in this retrospective chart review. Patient demographic, histologic, germline and somatic genetic testing, treatment duration, toxicity, and outcome data were collected. Clinical, pathological, and molecular characteristics were reported using descriptive statistics. Response rate by RECIST criteria, progression free survival, and clinical benefit rate were calculated. Clinical benefit rate was defined as patients who achieved complete response, partial response, or at least 6 months of stable disease as a result of therapy divided by the total number of patients in this cohort.

## 3 Results

### 3.1 Patient population

**Fig. 1:**
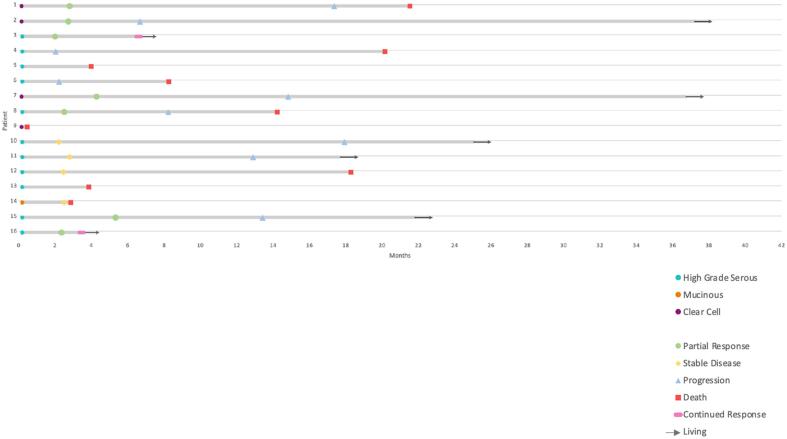
Swimmer’s plot detailing tumor histology, treatment outcome and follow up of patients who received pembrolizumab and lenvatinib combination therapy.

Sixteen patients treated with pembrolizumab and lenvatinib for recurrent MSS/pMMR ovarian cancer between January 2020 and April 2024 were identified by retrospective chart review. Pembrolizumab 200 mg was administered intravenously every 3 weeks with daily oral lenvatinib. Eleven patients had high-grade serous, four had clear cell, and one had mucinous histology. All but one patient had an initial diagnosis of FIGO stage III/IV disease (n = 15, 93.75 %) (Table 1, Fig. 1) Two patients had a germline BRCA mutation, and three additional patients had tumors that were homologous recombination deficient (HRD) including one with a somatic BRCA mutation; all were previously treated with a PARP inhibitor. Of the patients who underwent next generation sequencing (n = 11), all were MSS stable with low tumor mutational burden (TMB).

**Table 1:** Patient demographics and tumor characteristics.

Patient	Age	Race	Ethnicity	Histology	Stage at Diagnosis	Germline	MSI	TMB (Muts/Mb)	LOH	HRD	Somatic Gene Mutations
1	62	Korean	Non-Hispanic	OCCC	IIIC	Negative	NA	NA	NA	NA	NA
2	40	Hispanic	Puerto Rican	OCCC	IC3	Negative	Stable	1	5 %	Negative	MYC, CCND3, LYN, RAD21D, VEGFA amplification
3	72	White	Other	HGSC	IIIC	BRIP1	ND	ND	ND	ND	BRIP1
4	66	White	Non-Hispanic	HGSC	IIIC	Negative	Stable	5	16 %	Positive	CCND3 amplification
5	74	African American	Non-Hispanic	HGSC	IIIB	NA	NA	NA	NA	NA	NA
6	48	White	Non-Hispanic	HGSC	IVB	BRCA2	ND	ND	ND	ND	BRCA2, TP53
7	73	White	Non-Hispanic	OCCC	IIIB	NA	Stable	4	5.50 %	Negative	ARID1A, PIK3CA, BCL2L2 amplification, KDM6A, KDR, TET2
8	77	White	Non-Hispanic	HGSC	IVB	Negative	Stable	2NA	7 %	Negative	PIK3CA, TP53
9	60	White	Non-Hispanic	OCCC	IIIC	Negative	Stable	2	5.30 %	Negative	ARID1A, PPP2R1A
10	73	White	Non-Hispanic	HGSC	IIIC	Negative	Stable	9	9.40 %	Negative	AURKA, GNAS, and ZNF217 amplification, FANCL, KDM6A loss, TP53
11	59	Hispanic	Puerto Rican	HGSC	IIIC	Negative	Stable	4	23.30 %	Positive	RB1 loss, TP53
12	58	White	Non-Hispanic	HGSC	IIIA	BRIP1	Stable	4	NR	Negative	TP53
13	73	African American	Non-Hispanic	HGSC	IIIC	NA	NA	NA	NA	NA	NA
14	41	Asian Indian	Other	MOA	IVB	NA	Stable	4.1	NA	NA	GNAS, IL7R, PBRM1, PTPRS, TGFBR2
15	67	White	Non-Hispanic	HGSC	IIIC	BRCA1	Stable	1	20.50 %	Positive	BRCA1, MYC, PIK3CA, PRKCI, amplification, TP53
16	56	White	Non-Hispanic	HGSC	IIIC	Negative	Stable	2	0	Positive	BRCA1, MYC, TP53

HGSC = high-grade serous carcinoma; MOA = mucinous ovarian adenocarcinoma; OCCC = ovarian clear cell carcinoma; CPS = combined positive score; MSI = microsatellite instability; TMB = tumor mutational burden; LOH = loss of heterozygosity; HRD = homologous recombination deficiency; NA = not performed; ND = could not be determined; NR = not reported.

### 3.2 Treatment characteristics and response

Eighty-one percent of patients had platinum resistant disease and a median of 3 prior lines of therapy (Table 2). The majority of patients (62.5 %) had received three or more prior lines of therapy. In accordance with NCCN guidelines, all received prior platinum therapy and many were exposed to bevacizumab; those eligible for parp inhibitors received one and no patients were previously treated with an immunotherapy agent. Of the three platinum sensitive patients, two did not tolerate repeat platinum therapy and one opted to proceed with immunotherapy following a discussion of therapeutic options.

**Table 2:** Treatment details and outcomes.

Patient	Previous lines of therapy	Platinum resistant disease	Number of P/L cycles administered	Response to P/L	PFS (months)
1	1	Yes	15	PR	16.9
2	1	Yes	8	PR	6.4
3	3	Yes	9	PR	6.3
4	5	Yes	3	Progression	1.8
5	5	No	1	Non-evaluable	3.7
6	3	No	3	Progression	2.0
7	1	Yes	19	PR	14.4
8	2	Yes	9	PR	7.9
9	2	Yes	1	Non-evaluable	0.3
10	4	No	6	SD	17.5
11	6	Yes	16	SD	12.5
12	5	Yes	5	SD	17.8
13	3	Yes	1	Non-evaluable	3.6
14	1	Yes	3	SD	2.6
15	6	Yes	16	PR	13.0
16	3	Yes	5	PR	3.2
P/L = pembrolizumab and lenvatinib; PFS = progression-free survival; PR = partial response; SD = stable disease					

Three patients discontinued therapy after one cycle, unrelated to drug toxicity, and were considered non-evaluable for treatment response. Of the 13 evaluable patients, 54 % (7of 13) had a partial response and 31 % (4 of 13) had stable disease after 3 cycles. The clinical benefit rate was 69 % (11 of 16). Of the nine evaluable high grade serous patients, seven (78 %) had either a partial response or stable disease. All three evaluable patients with clear cell histology exhibited a partial response. In patients with a partial response or stable disease, the median time to progression was 12.5 months with a PFS of 7.9 months for all evaluable patients.

Treatment related toxicities for each patient are described in Table 3. Thirteen patients (81 %) experienced at least one treatment-related adverse effect. Of the three patients who did not have any adverse effects documented, two only received one cycle prior to transitioning to hospice or dying due to their disease. The most common toxicities were diarrhea (38 %), hypothyroidism (19 %), and rash (19 %) (Table 4). One patient had a treatment interruption due to worsening renal dysfunction. However, she ultimately discontinued treatment due to disease progression (Table 3). Diarrhea was the most common grade 3 adverse event. A total of 6 patients had treatment held temporarily due to toxicity and no patients experienced grade 4 toxicity (Table 4). At the time of data analysis, two patients remained on treatment. Four patients required lenvatinib dose reductions, although it should be noted that the majority of patients had a starting dose of 10 mg daily (n = 13, 81.25 %).

**Table 3:** Treatment dosing and adverse effects.

Patient	Starting dose	Dose reduction	Toxicity	Reason for Discontinuation
1	20 mg	Yes	Diarrhea, creatinine increased	Progression
2	10 mg	No	Rash, pruritus	Progression
3	10 mg	No	Diarrhea	Active Treatment*
4	10 mg	Yes	Abdominal pain	Progression
5	10 mg	No	None	Hospice
6	10 mg	No	Nausea, vomiting, diarrhea, pelvic pain	Progression
7	10 mg	No	Arthralgias, fatigue, headache, hypertension, diarrhea, proteinuria, hypothyroidism	Progression
8	20 mg	Yes	Diarrhea	Progression
9	10 mg	No	None	Death
10	10 mg	Yes	None	Progression
11	10 mg	No	Diarrhea, blisters, rash, hypothyroidism	Progression
12	20 mg	No	Fatigue, creatinine increased	Progression
13	10 mg	No	Arthralgias, hyperglycemia	Patient Preference
14	10 mg	No	Nausea, vomiting, tremors	Bowel Perforation
15	10 mg	No	Rash, cellulitis, hypothyroidism	Progression
16	10 mg	No	Arthralgias, rash	Active Treatment*

**Table 4:** Treatment-related adverse events and outcomes.

Any treatment-related adverse events	Any grade	Grade 3^a^	Treatment delay
Diarrhea	6 (38)	3 (19)	3 (19)
Creatinine increased	2 (13)	0	1 (6)
Rash	3 (19)	0	0
Pruritis	1 (6)	0	0
Abdominal pain	1 (6)	0	0
Nausea	2 (13)	0	0
Vomiting	2 (13)	0	0
Pelvic pain	1 (6)	0	0
Arthralgia	2 (13)	0	0
Fatigue	2 (13)	0	0
Headache	1 (6)	0	0
Hypertension	1 (6)	1 (6)	0
Hypothyroidism	3 (19)	0	0
Blisters	1 (6)	0	0
Hyperglycemia	1 (6)	1 (6)	1 (6)
Tremor	1 (6)	0	0
Proteinuria	1 (6)	0	1 (6)
All data are n (%) ^a^ No grade 4 toxicities reported			

## 4 Discussion

Our retrospective study provides support for the development of prospective studies to evaluate the use of pembrolizumab and lenvatinib in recurrent platinum resistant ovarian cancer. In our cohort of 13 evaluable patients, over half of the patients had a partial response and another third had stable disease, which translated into a clinically meaningful and sustained benefit for the majority of the patients in the study. In patients with a partial response or stable disease, the median time to progression was 12.5 months in responders, and in all evaluable patients the PFS of 7.9 months is worthy of further study. Based on our results, treatment with combination pembrolizumab and lenvatinib is a safe and potentially beneficial regimen for pMMR recurrent platinum resistance ovarian cancer including clear cell carcinomas.

Until recently, management options for recurrent platinum resistant ovarian cancer were limited to single agent cytotoxic therapy with the potential addition of bevacizumab. The phase 3 AURELIA trial reported a statistically significant improvement in ORR of 27.3 % and PFS of 6.7 months with the addition of bevacizumab to investigator choice chemotherapy (Pujade-Lauraine et al., 2014). An exploratory analysis demonstrated an improvement in PFS to 9.6 months with weekly paclitaxel and bevacizumab.

The advent of targeted therapy has improved the treatment landscape for platinum resistant ovarian cancer patients. For those with high folate receptor alpha (FRα) expression (≥75 % of cells with ≥ 2 + staining intensity), mirvetuximab soravtansine, an antibody-drug conjugate (ADC), demonstrated an ORR of 42.3 % with a median PFS of 5.6 months in the confirmatory MIRASOL trial (Moore et al., 2023). However, only a limited number of ovarian cancer patients will qualify for this therapy based upon FDA guidelines. Immunogen, the manufacturer of mirvetuximab soravtansine, has approximated that 35–40 % of ovarian cancer patients express high levels of FRα (Immunogen, 2022). Given that a significant proportion of patients are not candidates for this treatment, more options are needed.

Trastuzumab deruxtecan, another ADC, received accelerated tumor-agnostic approval for cancers expressing 3 + HER2 immunohistochemistry (IHC) in April 2024 based on the series of DESTINY trials (Kemp, 2024). Eleven ovarian cancer patients with 3 + IHC in the DESTINY Pan Tumor 02 trial had a 63.6 % response rate and a median PFS of 12.5 months (Meric-Bernstam et al., 2024). However, a 2007 study evaluating HER2 staining in 320 ovarian cancer samples identified only 15 (4.7 %) tumors with 3 + staining and 26 tumors (8.1 %) with 2 + staining (Tuefferd et al., 2007). NCCN guidelines also include patients with 2 + IHC. Data from DESTINY indicates an ORR of 45 % and a PFS of 5.9 months for the 2+/3 + IHC cohort (Meric-Bernstam et al., 2024). With approximately 85 % of ovarian cancers patients not eligible for use of ADC targeted therapy and the less than 6-month median PFS associated with use of these drugs, the need for additional effective management options remains acute.

Studies evaluating the use of single agent immune checkpoint inhibitors (ICI) in recurrent platinum resistant ovarian cancer have not shown significant clinical benefit (Disis et al., 2019, Brahmer et al., 2012, Varga et al., 2019). For example, in a phase 1b trial of PD-L1-positive metastatic ovarian cancer patients, only 12 % of those treated with single agent pembrolizumab had a response and 27 % achieved stable disease (Varga et al., 2019). Another phase 1b study of avelumab for previously treated recurrent or refractory ovarian cancer patients reported an ORR of 9.6 % (Disis et al., 2019). Furthermore, in the phase 2 KEYNOTE-100 trial, treatment with pembrolizumab monotherapy demonstrated an ORR of 8 % in patients who received ≤2 prior lines of therapy and 10 % in those who received 3 to 5 prior lines of therapy (Matulonis et al., 2019). In the primary setting, treatment responses with ICIs have also been limited (Monk et al., 2021, Moore et al., 2021).

Clinical trials have explored ways to augment the immune system by combining ICI with either chemotherapy, another ICI, antiangiogenic therapy, or PARP inhibitors. Angiogenesis is crucial for cancer growth and metastasis. VEGF is known to be highly expressed in nearly all EOC (van der Bilt, et al., 2012, Yamamoto et al., 1997). Preclinical studies have shown that lenvatinib, a multiple receptor tyrosine kinase inhibitor, has immunomodulatory activity which improves T-cell activation and reduces tumor-associated macrophages, resulting in enhanced antitumor activity when combined with anti-PD-1 antibody (Kato et al., 2019, Kimura et al., 2018, Tran et al., 2023). This has led to the FDA approval for the combination of pembrolizumab/lenvatinib in MSS/pMMR endometrial cancer and renal cell carcinoma.

While combination treatment with pembrolizumab and lenvatinib is not FDA approved for use in ovarian cancer, recently published data from the LEAP-005, a phase 2, multicohort, open-label study of pembrolizumab/lenvatinib as fourth-line therapy in 31 patients with advanced ovarian cancer demonstrated a 35 % objective response rate (ORR) and 42 % had stable disease (González-Martín et al., 2024). Median PFS was 6.2 months and median OS was 21.3 months (González-Martín et al., 2024). Preliminary results from an ongoing phase 2 study by Bauer et al evaluating the effectiveness of pembrolizumab and lenvatinib in recurrent platinum sensitive ovarian cancer demonstrated RECIST 1.1 response in 50 % of the patients, 8 % of which were complete responses (Bauer et al., 2024). Case reports examining pembrolizumab and lenvatinib therapy in recurrent, platinum resistant ovarian clear cell carcinoma (OCCC) have also demonstrated favorable clinical responses in this population (Calo et al., 2023, McNamara et al., 2023). Our positive study adds to this body of literature providing further justification for the pursuit of prospective data and allocation of resources into higher level evidence. While prospective randomized control data needed to gain FDA approval for use of pembrolizumab/lenvatinib in ovarian cancer is not yet available, based on our study and published results, it is an appropriate option to offer patients.

Use of ICIs and other immunotherapy is associated with a spectrum of side effects, different from traditional cytotoxic chemotherapy. Safety profiles and treatment-related adverse events (AEs) must be closely monitored when treating patients with these agents. Immune related AEs vary based on class and dose of treatment. In the LEAP-005 study, 94 % of patients experienced treatment-related AEs with seven patients (23 %) having to discontinue the study drug (González-Martín et al., 2024). In the phase 2 study by Bauer, treatment related AEs occurred in all 24 patients with discontinuation by 3 (Bauer et al., 2024). In our cohort one patient had treatment interruption due to suspected immune mediated nephrotoxicity; however, this patient had pre-existing renal dysfunction and ultimately treatment was discontinued due to disease progression. While the majority of our patients experienced at least one toxicity, no patients discontinued this treatment regimen due to toxicity. Side effects were manageable with standard therapies and patients who had treatment held due to toxicity were ultimately able to resume treatment after resolution of symptoms. It is important to note; however, given the retrospective nature of this review, toxicities were not monitored using a standardized protocol as would be done in a prospective trial. As such, toxicity information was obtained via chart review and details were subject to the documentation of providers. Also worth noting; in our heavily pretreated population, the majority of our patients initiated lenvatinib at 10 mg daily with the plan for dose escalation as tolerated. The majority of patients remained at 10 mg daily with 4 requiring dose reduction. This could explain the overall low toxicity rates seen in our study. Even though 13 out of 16 patients were treated with lenvatinib doses of 10 mg or less, our data suggest that lower doses still provide clinical benefit.

## 5 Conclusion

As the treatment landscape of cancers shifts towards more targeted approaches and immunotherapy, our study adds to the growing body of literature supporting the use of combination pembrolizumab and lenvatinib for the treatment of recurrent, platinum resistant MSS/pMMR ovarian cancer. The meaningful responses and manageable toxicity demonstrated in this trial supports the ongoing study of pembrolizumab and lenvatinib in the treatment of platinum resistant ovarian cancer. It should also be noted that meaningful treatment responses occurred despite the majority of patients being treated with 10 mg of lenvatinib instead of the typical starting dose of 20 mg. Although our study is descriptive, the clinical benefit and PFS reported here suggests this regimen is at least as active and more broadly applicable than available options including the addition of bevacizumab to single agent chemotherapy, mirvetuximab, and trastuzumab deruxtecan.

## CRediT authorship contribution statement

**Helen Toma:** Writing – review & editing, Writing – original draft, Investigation, Data curation, Conceptualization. **Rebeca Kelly:** Writing – review & editing. **Chelsea Katz:** Writing – review & editing, Data curation. **Hannah Hong:** Writing – review & editing. **Hannah Diasti:** Writing – review & editing, Visualization, Data curation. **David P. Warshal:** Writing – review & editing. **Lauren Krill:** Supervision.

## Declaration of competing interest

The authors declare that they have no known competing financial interests or personal relationships that could have appeared to influence the work reported in this paper.
